# Detecting early memory changes in preclinical Alzheimer's disease using TabCAT favorites test: Data from the European Prevention of Alzheimer's Disease (EPAD) cohort

**DOI:** 10.1002/alz.71035

**Published:** 2026-02-13

**Authors:** Anna Brugulat‐Serrat, Elena Tsoy, Gonzalo Sánchez‐Benavides, Marta Milà‐Alomà, Leslie S. Gaynor, Oriol Grau‐Rivera, Juan Domingo Gispert, Joel H Kramer, Katherine L Possin, for the European Prevention of Alzheimer's Disease (EPAD) Consortium

**Affiliations:** ^1^ Barcelonaβeta Brain Research Center Pasqual Maragall Foundation Barcelona Spain; ^2^ IMIM (Hospital del Mar Medical Research Institute) Barcelona Spain; ^3^ Centro de Investigación Biomédica en Red de Fragilidad y Envejecimiento Saludable (CIBERFES) Madrid Spain; ^4^ Global Brain Health Institute San Francisco California USA; ^5^ Faculty of Medicine University of Vic‐Central University of Catalonia (UVic‐UCC) Catalonia Spain; ^6^ Department of Neurology University of California San Francisco San Francisco California USA; ^7^ Northern California Institute for Research and Education San Francisco California USA; ^8^ Department of Radiology and Biomedical Imaging University of California San Francisco San Francisco California USA; ^9^ Division of Geriatric Medicine Department of Medicine Vanderbilt University Medical Center Nashville Tennessee USA; ^10^ Centro de Investigación Biomédica en Red Bioingeniería Biomateriales y Nanomedicina, (CIBER‐BBN) Madrid Spain

**Keywords:** computerized cognitive assessment, early detection, episodic memory, preclinical Alzheimer's disease

## Abstract

**INTRODUCTION:**

Sensitive memory paradigms may allow the detection of subtle memory changes associated with early Alzheimer's pathology in individuals without established clinical symptomatology.

**METHODS:**

We explored the cross‐sectional association between performance on Tablet‐based Cognitive Assessment Tool (TabCAT) Favorites, a brief computerized memory test, with cerebrospinal fluid AT status (A for amyloid‐β and T for phosphorylated tau) and its discriminative validity in 727 clinically asymptomatic participants from the European Prevention of Alzheimer's Disease (EPAD) Longitudinal Cohort Study. Episodic memory was also evaluated with the Repeatable Battery for the Assessment of Neuropsychological Status Delayed Memory Index (RBANS‐MI).

**RESULTS:**

Compared to A‐T‐ individuals, poorer TabCAT Favorites Total Correct (Favorites‐TC) cross‐sectional performance was associated with an increased likelihood of A+T+ status, but not A+T‐ status. There were no significant associations between AT status and RBANS‐MI. Among individuals with low Favorites‐TC performance, AT status predicted progression on the Clinical Dementia Rating > 0.

**DISCUSSION:**

Favorites‐TC is a sensitive measure for the early detection of cognitive changes in the early stages of the AD *continuum*.

**Highlights:**

- We explored Tablet‐based Cognitive Assessment Tool (TabCAT) Favorites scores and cerebrospinal fluid (CSF) AT status (A for amyloid‐β and T for phosphorylated tau) in asymptomatic individuals.
- Poorer Favorites performance linked to higher A+T+ likelihood.
- TabCAT Favorites is a sensitive tool for detecting early cognitive changes in Alzheimer's disease (AD).

## 1 BACKGROUND

There is growing evidence that Alzheimer's disease (AD) pathological changes, namely amyloid‐β (Aβ) and phosphorylated tau (p‐tau) accumulation, precede the onset of clinically significant cognitive symptoms^1^. Understanding the relationship between AD biomarkers and cognitive performance in cognitively unimpaired (CU) individuals represents a fundamental step toward early detection and intervention strategies. However, the link between subtle cognitive decline and preclinical biomarker stages remains unclear, and traditional neuropsychological assessments may not be sufficiently sensitive for early detection.^2^, ^3^

Episodic memory decline is the hallmark early cognitive symptom of AD^4^, and prior evidence indicates that early Aβ pathology is associated with subtle memory changes in otherwise asymptomatic individuals without clinically significant impairments established via standard diagnostic procedures.^5^ Indeed, prior studies in clinically asymptomatic individuals using sensitive episodic memory tests found significant associations of AD biomarker burden with longitudinal memory decline.^3^, ^6^, ^7^, ^8^ Thus, sensitive memory paradigms may allow for early detection of individuals at‐risk for AD, tracking the progression of subtle memory changes, and enhancing identification of candidates for clinical trials targeting preclinical stages of the disease process.^2^ However, research on cognitive measures capable of detecting AD biomarker burden in clinically asymptomatic individuals cross‐sectionally remains limited.^9^, ^10^

In this study, we examined whether performance on Favorites, a computerized test of associative memory, was cross‐sectionally associated with AD biomarker status in a multinational sample of clinically asymptomatic older adults from the European Prevention of Alzheimer's Dementia Longitudinal Cohort Study (EPAD LCS). Favorites is a 5‐minute tablet‐based test programmed in the Tablet‐based Cognitive Assessment Tool (TabCAT) software platform (University of California, San Francisco) and has been previously shown to exhibit high sensitivity to detecting mild cognitive impairment^11^, neuroanatomical validity^11^, and significant associations with Aβ and tau burden on positron emission tomography (PET) in cognitively impaired samples.^12^

**RESEARCH IN CONTEXT**

1. **Systematic review**: We reviewed literature on the use of memory tasks to detect subtle cognitive changes in preclinical Alzheimer's disease (AD) in PubMed using search terms “amyloid,” “tau,” “preclinical AD,” and “subtle memory decline.”
2. **Interpretation**: Our findings showed that the Tablet‐based Cognitive Assessment Tool (TabCAT) Favorites is a sensitive measure for detection of cognitive changes in earlier stages of the AD *continuum*, representing a valuable alternative to traditional episodic memory tests for clinical and research applications.
3. **Future directions**: Replication of the findings in independent cohorts and diverse populations is needed.

## 2 METHODS

### 2.1 Participants

**FIGURE 1:**
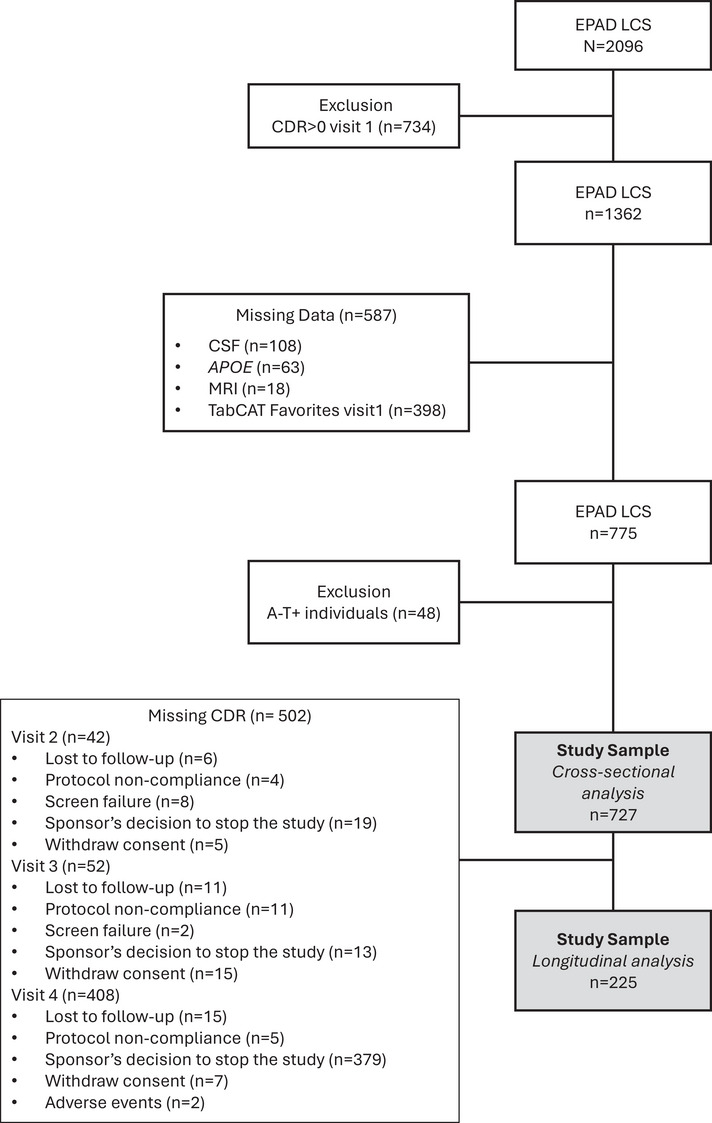
Participant selection flowchart.

Data for this study were obtained from the EPAD LCS V:IMI data set (doi:10.34688/epadlcs_v.imi_20.10.30).^13^, ^14^ In brief, EPAD LCS (www.clinicaltrials.gov Identifier: NCT02804789) is a prospective, multicenter, pan‐European longitudinal cohort study with participants recruited across 21 European sites from May 2016 to December 2019 as previously described.^15^ We included data from 727 clinically asymptomatic participants, defined as having Clinical Dementia Rating (CDR) Global Score = 0, who completed TabCAT Favorites, structural neuroimaging, cerebrospinal fluid (CSF), and apolipoprotein E (*APOE*) genotyping studies during the baseline visit (Figure 1). Our aim was to identify participants on the AD *continuum*, thus participants in the A‐T+ (A for amyloid‐β and T for phosphorylated tau) group (*n* = 48) were excluded from this study; however, a sensitivity analysis with A‐T+ group were performed (Table S1). A subset of 225 individuals (30.9%) with complete CDR data across four longitudinal visits were analyzed to assess the prognostic value of TabCAT Favorites total correct (Favorites‐TC). The remaining 502 participants were excluded from longitudinal analyses (Figure 1) due to loss to follow‐up (*n* = 32), protocol non‐compliance (*n* = 20), screen failure (*n* = 10), sponsor's decision to stop the study (*n* = 411), withdrawal of consent (*n* = 27), and adverse events (*n* = 2). All participants provided informed consent, and local ethical approval was obtained from ethics committees specific to each research site. All procedures were conducted in accordance with the ethical standards of the Helsinki Declaration.

### 2.2 Measures and procedures

#### 2.2.1 Cognitive tests

The EPAD cognitive assessment data was collected in accordance with a standardized protocol as described previously.^16^, ^17^ Episodic memory was evaluated with the Favorites‐TC^11^ and the Repeatable Battery for the Assessment of Neuropsychological Status Delayed Memory Index (RBANS‐MI).^18^ Favorites‐TC is a 5‐minute test of associative memory administered by a trained neuropsychologist where participants are asked to remember people's faces and their favorite food and animal. On each of the two learning trials, four different faces are shown twice, once with a favorite food and once with a favorite animal, creating eight face‐word associations in total. Each pair was displayed for five seconds in a pseudorandom order. Following each learning trial, the faces were presented individually, and participants were asked to recall both the food and animal associated with each face. A delayed recall trial was administered after a 10‐minute interval. The primary performance metric is Favorites‐TC, which is the sum of raw scores across two immediate trials and one 10‐minute delay trial (range: 0‐24). Favorites‐TC was administered in local languages after undergoing linguistic adaptation procedures, including forward and backward translations by professional interpretation services.^19^, ^20^ RBANS‐MI is a composite score based on three memory tests that take 10 min to complete: the recall of a previously learned word list, a short story, and a geometric figure.^18^ RBANS was administered in local languages using previously translated and validated versions, which were developed by the test publisher or local research teams independent of the EPAD standard procedures.

#### 2.2.2 Neuroimaging

Brain magnetic resonance imaging (MRI) scans were performed with standardized acquisition protocols, including 3D‐T1, 3D‐FLAIR, 2D‐T2, and 2D‐T2* sequences. The images were centrally evaluated by experienced raters blinded to the clinical and neuropsychological data. Regional gray matter volumes were determined on 3D‐T1 weighted images using a segmentation process based on atlas‐propagation with the Learning Embeddings for Atlas Propagation (LEAP) framework.^21^ Total intracranial volume ‐adjusted hippocampal volumes (HVa) were calculated as the residuals of a linear regression using TIV as independent variable. HVa is a well‐established measure of neurodegeneration in AD^22^, ^23^ and an indicator of disease severity/stage.^24^

#### 2.2.3 *APOE* genotyping

*APOE* genotyping was performed using TaqMan Genotyping Assays of blood samples analyzed in a single laboratory at the University of Edinburgh using QuantStudio 12KL Flex. Participants were classified as ε*4* carriers (one or two alleles) or ε*4* non‐carriers.

#### 2.2.4 CSF biomarker measurements and cutoffs

The biomarker data available in the EPAD protocol include CSF measures of Aβ_42_, total tau (t‐tau), and p‐tau. CSF collection was performed using a harmonized pre‐analytical protocol. Analyses were performed using the fully automatized Roche Elecsys System in a single laboratory (University of Gothenburg)^13^. Concentrations of Aβ_42_, t‐tau, and p‐tau were determined according to the manufacturer's instructions. Aβ positivity (A+) was defined as Aβ_42 _< 1000pg/mL and tau positivity (T+) as p‐tau181 > 27pg/mL in accordance with previously established cutoffs.^25^ Participants were classified into AT groups based on these biomarker thresholds, resulting in four groups: A–T–, A+T–, A–T+, and A+T+.

#### 2.2.5 Statistical analyses

The AT groups were compared on demographic, neuropsychological, neuroimaging, and genetic characteristics using one‐way analyses of variance (ANOVAs) for continuous variables or χ‐squared tests for categorical variables. To examine the associations between neuropsychological measures and AT stages, we conducted general linear models with raw scores on Favorites‐TC or RBANS‐MI as outcomes and AT groups as predictors. Covariates included age, sex, education level (elementary = 7‐11 years, secondary = 12‐14, graduate = 15‐17, postgraduate = 18‐20), and *APOE*‐ ε4 status (carriers and non‐carriers). Next, we performed two independent multinomial logistic regression models for each neuropsychological measure (Favorites‐TC and RBANS‐MI) to examine whether these measures could predict AT group classifications. In the first set of models, AT group was set as the dependent variable and Favorites‐TC and RBANS‐MI were included as predictors. Covariates included age, sex, education level, and *APOE*‐ ε4 status. In secondary models, we additionally covaried for HVa to explore the potential role of neurodegeneration in these associations. Odds ratios (ORs) were derived from these models to measure the strength of the association between memory performance and AT group classification. Values > 1 indicate an increased likelihood of being classified in a higher AT stage, while values < 1 indicate lower likelihood.

Next, we performed receiver operating characteristic (ROC) analyses for each memory measure to obtain the area under the curve (AUC) for discriminating between A‐T‐ and A+T+ groups. DeLong's test was used to compare AUCs for the different tests. Optimal cutoffs were identified based on the Youden's J Index (YI)^26^, and the overall percentage agreement (OPA; “accuracy”) at the optimal cutoff was computed. The YI represents the optimal balance between sensitivity and specificity with values closer to 1 indicating better discriminative ability of the test in distinguishing between AT groups. We also examined the prognostic utility of the optimal cutoff across AT groups for CDR progression across four longitudinal visits (mean of 1.91 [SD 0.34] follow‐up years from baseline visit) in a subset of 225 individuals, using Cox proportional hazards models adjusted by age at first visit, sex, and educational level. The significance level was set at *p *< 0.05. All analyses were performed in SPSS IBM v28.0 and R v3.6.0.

## 3 RESULTS

### 3.1 Participants' characteristics

Table 1 summarizes demographic, biomarker, genetic, volumetric, and cognitive performance characteristics of 727 study participants by AT staging. These comparisons were based on raw (unadjusted) cognitive scores. A+T+ group was significantly older (*p *< 0.001) and more likely to be *APOE*‐ ε4 carriers (*p *< 0.001). A+T+ participants also performed significantly worse on Favorites‐TC (*p *< 0.001), but not RBANS‐MI (*p *= 0.262), compared to the other groups.

**TABLE 1:** Descriptive characteristics of the study participants by AT staging.

Parameter	Total	A‐T‐	A+T‐	A+T+	*p*‐value
N, (%)	727	519 (71.4)	175 (24.1)	33 (4.5)	
Age, M (SD)	64.9 (6.8)	64.4 (6.3)	65.6 (6.8)^*^	70.6 (6.5)^*^, ^†^	<0.001^a^
Female, N (%)	425 (58.5)	313 (60.3)	95 (54.3)	17 (51.5)	0.267
Education, N (%)					0.050
Elementary	162 (22.3)	103 (19.8)	44 (25.1)	15 (45.4)	
Secondary	172 (23.6)	135 (26.0)	33 (18.9)	4 (12.1)	
Graduate	308 (42.4)	224 (43.2)	71 (40.6)	13 (39.4)	
Postgraduate	85 (11.7)	57 (10.9)	27 (15.4)	1 (3.0)	
Testing language, N (%)					0.207
Dutch	104 (14.3)	84 (16.2)	16 (9.1)	4 (12.1)	
English	180 (24.8)	120 (23.1)	50 (0.29)	10 (30.3)	
French	118 (16.2)	94 (18.1)	20 (11.4)	4 (12.1)	
Spanish	325 (44.7)	221 (42.6)	89 (50.9)	15 (45.4)	
*APOE*‐ ε4 carrier, N (%)	278 (38.2)	165 (31.8)	88 (50.3)^*^	25 (75.8)^*^, ^†^	<0.001^a^
CSF biomarkers, M (SD)					
Aβ_42_ (pg/mL)	1411.2 (599.8)	1677.0 (493.8)	754.8 (175.5)^*^	711.5 (200.1)^*^	<0.001^a^
p‐tau181 (pg/mL)	16.9 (5.7)	16.3 (3.9)	15.6 (5.1)^*^	34.3 (5.7)^*^, ^†^	<0.001^a^
Cognitive tests, M (SD)					
Favorites‐TC	15.4 (5.6)	15.6 (5.6)	15.4 (5.3)	11.8 (5.2)^*^, ^†^	<0.001^a^
RBANS‐MI	104.4 (12.3)	104.4 (12.3)	104.9 (14.6)	101.9 (14.6)	0.262
Neuroimaging, M (SD)					
Hippocampal volume (mm^3^)	4750.2 (854.9)	4754.9 (897.8)	4713.1 (761.5)	4871.9 (612.8)	0.278
Whole brain volume (mm^3^)	1112.5 (104.2)	1109.6 (104.8)	1122.7 (102.8)	1105.0 (102.6)	0.414

*Notes*: Data are expressed as mean (M) and standard deviation (SD) or percentage (%), as appropriate. One‐way ANOVA followed by Tukey corrected post hoc comparisons were used for age, education, CSF biomarkers, cognitive tests, and neuroimaging variables, and Pearson's χ2 tests to compare sex, testing language, and APOE‐ε4 status among AT groups.

*p*‐values indicate the AT group effects and are corrected for multiple comparisons using FDR approach.

Abbreviations: Aβ42, amyloid‐β 42; ANOVA, analysis of variance; APOE, apolipoprotein E; A/T, A for amyloid‐β and T for phosphorylated tau; CSF, cerebrospinal fluid; Favorites‐TC, Favorites Total Correct; FDR, false discovery rate; p‐tau, phosphorylated tau; RBANS‐MI, Repeatable Battery for the Assessment of Neuropsychological Status Delayed Memory Index.

^a^ Significant values.

Pairwise post hoc comparisons:

* *p* < 0.05 versus A‐T‐.

^†^ *p* < 0.05 versus A+T‐.

### 3.2 Associations between episodic memory and AT stages

**FIGURE 2:**
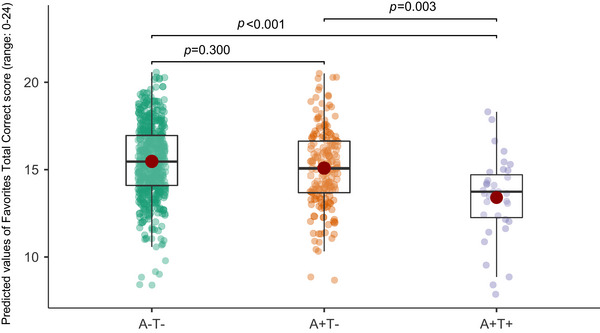
Dot and box plots depicting the predicted value of the Favorites‐TC Score in each of the AT groups covarying for age, sex, education, testing language, and *APOE*‐ ε4 status. A+T+ individuals (purple) showed significantly worse Favorites‐TC performance compared to those in the A‐T‐ (green; *p *< 0.001) and A+T‐ (orange; *p *= 0.003) groups. *p‐*Values are based on the pairwise Bonferroni *post hoc* comparisons. APOE, apolipoprotein E; A/T, A for amyloid‐β and T for phosphorylated tau; Favorites‐TC, Tablet‐based Cognitive Assessment Tool Favorites Total Correct.

In multinomial regression models with A‐T‐ as reference group (Table 2, Figure 2, Table S2), we found that poorer Favorites‐TC performance was associated with an increased likelihood of A+T+ status (OR = 0.92, 95% confidence interval [CI] = 0.85–0.98, *p *= 0.016), but not with A+T‐ status (OR = 1.0, 95% CI = 0.97–1.04, *p *= 0.757). In contrast, we did not find significant associations between RBANS‐MI performance with either A+T‐ status (OR = 1.00, 95% CI = 0.94‐1.02, *p *= 0.539) or A+T+ status (OR = 1.00, 95% CI = 0.97–1.03, *p *= 0.844). Greater age and presence of an *APOE*‐ ε4 allele were also significantly associated with greater likelihood of A+T‐ and A+T+ status (Table S1). When HVa was included as a covariate (Table S2), the associations between Favorites‐TC performance and A+T+ status were slightly attenuated but remained significant (OR = 0.99, 95% CI = 0.85–0.99, *p *= 0.033). In sensitivity analyses including A+T‐ individuals, these results remained significant (Table S3). Individuals in the A+T+ group also showed significantly worse Favorites‐TC performance compared to those in the A+T‐ group in models covarying and not covarying for HVa (*p *= 0.003 and *p *= 0.014, respectively; Figure 2).

**TABLE 2:** Associations between Favorites‐TC and RBANS‐MI and AT stages.

	A+T‐				A+T+			
Ref. group = A‐T‐	**B**	**OR**	**95% CI of OR**	***p*‐value**	**B**	**OR**	**95% CI of OR**	***p*‐value**
Favorites‐TC	0.00	1.00	0.97 – 1.04	0.757	−0.09	0.92	0.85 – 0.98	0.016^*^

	**A+T‐**				**A+T+**			
Ref. group = A‐T‐	**B**	**OR**	**95% CI of OR**	***p*‐value**	**B**	**OR**	**95% CI of OR**	***p*‐value**
RBANS‐MI	0.01	1.00	0.9 – 1.02	0.539	0.01	1.00	0.97 – 1.03	0.844

*Notes*: The results are covaried for age, sex, education level, and *APOE*‐ ε4 status.

Abbervations: A/T, A for amyloid‐β and T for phosphorylated tau; APOE, apolipoprotein E; CI, confidence interval; Favorites‐TC, Tablet‐based Cognitive Assessment Tool Favorites Total Correct; OR, odds ratio; RBANS‐MI, Repeatable Battery for the Assessment of Neuropsychological Status Delayed Memory Index.

* *p* < 0.05

### 3.3 Optimal episodic memory cutoffs

**FIGURE 3:**
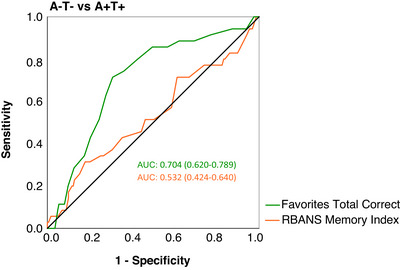
ROC curves distinguishing A+T+ and A‐T‐ groups based on Favorites‐TC and RBANS‐MI performance. A/T, A for amyloid‐β and T for phosphorylated tau; Favorites‐TC, Tablet‐based Cognitive Assessment Tool Favorites Total Correct; RBANS‐MI, Repeatable Battery for the Assessment of Neuropsychological Status Delayed Memory Index; ROC, receiver operating characteristics.

Favorites‐TC had a higher AUC (0.704; 95% CI 0.620‐0.789) in distinguishing A+T+ from A‐T‐ groups than RBANS‐MI (0.532; 95% CI 0.424‐0.640; Figure 3). DeLong's test revealed that the difference between AUCs was significant (*p *= 0.001; 95% CI 0.067‐0.278). The optimal cutoff according to the maximum YI value was ≤13 on Favorites and had a sensitivity of 71% and a specificity of 69% with an OPA of 71% (Table 3). Tabular results for sensitivity and specificity based on Favorites performance are reported in Table S4. Based on the Favorites‐TC optimal cutoff, 70.1% (*n* = 364) of A‐T‐ individuals had a Favorites‐TC > 13 (control group), 29.9% (*n* = 155) of A‐T‐ individuals had a Favorites‐TC≤13, and 69.7% (*n* = 23) of A+T+ individuals had a Favorites‐TC≤13.

**TABLE 3 alz71035-tbl-0003:** Sensitivity, specificity, and Youden's Index for the Favorites‐TC cutoffs

Cutoff	Sensitivity (%)	Specificity (%)	YI	PPV (%)	NPV (%)
≤13 (YI)	71.0	69.0	0.41 (Max)	82.1	96.7
≤9	28.6	80.0	0.16	79.8	95.2
≤8	20.0	85.0	0.10	75.0	95.1

Abbreviations: NPV, negative predictive value; PPV, positive predictive value; YI, Youden Index; Favorites‐TC, Tablet‐based Cognitive Assessment Tool Favorites Total Correct.

### 3.4 Prognostic utility of the Favorites‐TC cutoff

**FIGURE 4 alz71035-fig-0004:**
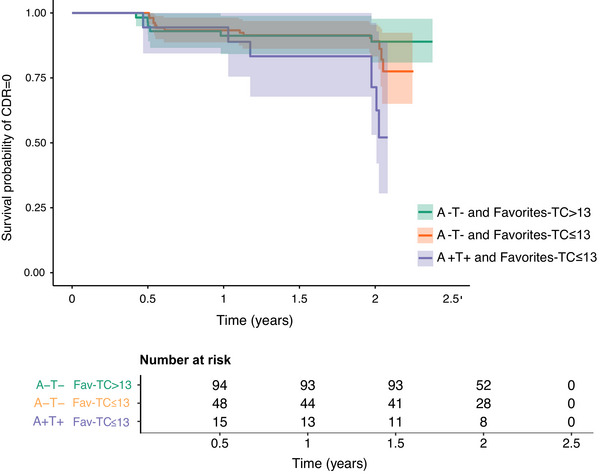
Progression to CDR > 0 for A‐T‐ and A+T+ groups by Favorites‐TC optimal cutoff. Survival curves are shown in green for A‐T‐ group with Favorites‐TC > 13, in orange for A‐T‐ group with Favorites‐TC≤13, and in purple for A+T+ group with Favorites‐TC≤13. Survival curves are adjusted for age, sex, and education level. A/T, A for amyloid‐β and T for phosphorylated tau; CDR, Clinical Dementia Rating; Favorites‐TC, Tablet‐based Cognitive Assessment Tool Favorites Total Correct.

The Favorites‐TC prognostic utility was assessed in a subset of 225 individuals who completed the CDR across four longitudinal visits. A+T+ individuals with Favorites‐TC≤13 showed a significantly faster progression to CDR > 0 compared to the control group (33.3%, *p *= 0.014) and A‐T‐ individuals with Favorites‐TC≤13 (14,6%, *p *= 0.014; Table 3, Figure 4). A+T+ individuals with a Favorites‐TC > 13 (*n* = 10) were not included in these analyses as no CDR progression was reported in this group over the study period. Regardless of the biomarker status, individuals with Favorites‐TC≤13 showed a significantly faster progression to CDR > 0 (*p *= 0.002) compared to individuals with Favorites‐TC > 13 (Figure S1).

## 4 DISCUSSION

In the present study, we examined the associations between cross‐sectional performance on TabCAT Favorites, a brief computerized associative memory test, with biomarker status and future clinical progression in a large multinational sample of clinically asymptomatic individuals.[Table alz71035-tbl-0003], [Fig alz71035-fig-0004]

Our results show that poorer performance on Favorites‐TC was significantly associated with the likelihood of CSF A+T+ status, but not A+T‐, in clinically asymptomatic individuals at baseline, after adjusting for age, sex, education, and *APOE*‐ε4 status. Specifically, clinically asymptomatic Aβ+ individuals with elevated levels of CSF p‐tau181 performed worse on Favorites‐TC than those with normal CSF p‐tau181 levels. The results remained significant after controlling for total intracranial volume adjusted hippocampal volume, suggesting that associative memory performance on Favorites‐TC is likely associated with early AD pathophysiology beyond hippocampal volumetric changes in clinically asymptomatic older adults.^27^, ^28^, ^29^, ^30^, ^31^ Several other studies have suggested that tau accumulation has a stronger association with cognitive performance compared to Aβ.^32^ Our findings contribute to this literature and support the notion that highly sensitive episodic memory measures can detect early p‐tau181 burden in Aβ+ clinically asymptomatic individuals, even cross‐sectionally.^33^

Favorites‐TC also demonstrated greater accuracy in discriminating A‐T‐ and A+T+ groups compared to the widely used RBANS‐MI. These results are consistent with prior literature supporting a particular sensitivity of associative memory and binding paradigms to AD‐related neuropathological changes.^34^, ^35^ We further found that A+T+ individuals performing below an empirically defined Favorites‐TC optimal cutoff showed a significantly faster clinical progression to CDR > 0, compared to the A‐T‐ individuals. These findings support the notion that Favorites‐TC performance is not only associated with AD signature biomarkers cross‐sectionally but also has the potential for clinical prognostic utility and risk stratification among clinically asymptomatic individuals. Our results align with previous evidence that associative learning tasks are valuable for identifying subtle cognitive difficulties in the preclinical stage of AD which may not be evident in standard memory assessments.^33^, ^36^, ^37^, ^38^ Overall, our findings contribute to the expanding body of literature on sensitive and accurate neuropsychological measures that can detect cognitive changes during the early stages of the AD continuum and help identify individuals who are at risk of clinical progression.

A particular strength of Favorites‐TC is its brevity (5‐minute administration time), making it highly feasible for wide‐scale implementation in busy clinical settings^11^, ^39^ and large research studies. In addition, given its digital nature, Favorites‐TC does not require administration by a trained specialist or manual scoring or data entry, thus enhancing its potential use in non‐specialty settings^11^. Overall, Favorites‐TC is a valuable test that is particularly suited for the early detection, monitoring, and disease staging in research settings and clinical trials involving asymptomatic individuals with underlying AD pathology. Its ability to identify early pathological changes associated with AD is especially helpful for stratifying participants in prevention trials and tracking cognitive decline over time. Moreover, the potential use of Favorites‐TC or similar tools in clinical settings may promote advancements in precision medicine and enable more targeted interventions in Alzheimer's research and therapies.^12^

Some limitations of this work should be considered. First, the EPAD LCS cohort is predominantly of European ancestry, with less than 1% of the sample representing other ethnicities. As a result, generalizations to the broader population should be made with caution. While the use of a multinational cohort is a strength of the study, replication of the findings presented here in independent cohorts would be valuable for validating and potentially extending the results to diverse populations. Encouragingly, Favorites‐TC has also been culturally adapted to multiple languages and geographies, as shown in this multinational study that included English, Dutch, Finnish, French, German, and Spanish versions, supporting its applicability across linguistically diverse populations.^40^, ^41^ This feature enhances the tool's potential, making cognitive testing more accessible and promoting greater diversity among participants in research and intervention studies. Digital assessments, however, may unintentionally exacerbate health disparities if they are found to exhibit poor validity in individuals with limited digital literacy or access to technology.^42^ Validation studies across diverse socioeconomic, educational, and cultural backgrounds will be essential to ensure that Favorites‐TC maintains its sensitivity and specificity across all populations. Additionally, our analyses included raw Favorites‐TC scores to derive an optimal cutoff, but future studies should develop demographically adjusted normative data on this measure to enhance its clinical interpretability across populations. Second, it should be noted that the AT status in this study was defined using CSF biomarkers, which are known to change earlier in the AD continuum than molecular neuroimaging biomarkers.^43^ Different operationalizations of the AT system have shown strong effects on category prevalence and predictions of future cognitive decline. Additionally, AT definition has implications for its interpretation in research studies, clinical trial design, and potentially in clinical practice. For example, prevention trials focusing on the very early AD stages may benefit from using CSF biomarkers, but prevention trials designed for the clinically symptomatic stages or assessments in clinical practice may benefit from using brain PET instead.^44^In this evolving biomarkers landscape, plasma biomarkers have gained significant attention as minimally invasive, cost‐effective, and accessible alternatives to CSF and PET measures.^45^ Future studies should examine whether the combination of sensitive memory tools and AD plasma biomarkers may enhance early detection and monitoring of AD pathology. Lastly, the presence of missing data in longitudinal analyses is a limitation and should be taken into account when interpreting the prognostic findings.

In conclusion, Favorites‐TC is a sensitive measure for early detection of cognitive changes in the earlier stages of AD *continuum*, representing a valuable alternative to traditional episodic memory tests for clinical and research applications. Our results show that brief cognitive measures can effectively indicate AD‐related changes, particularly when comprehensive neuropsychological assessments are impractical due to time, staffing, or cost constraints.^12^

## ACKNOWLEGDMENTS

The authors express their most sincere gratitude to the EPAD LCS participants. This work used data and/or samples from the EPAD project, which received support from the EU/EFPIA Innovative Medicines Initiative Joint Undertaking EPAD grant agreement n° 115736 and an Alzheimer's Association Grant [SG‐21‐818099‐EPAD]. AB‐S receives funding from Alzheimer's Association Clinician Scientist Fellowship Program (AACSF‐23‐1145154). ET received funding from the NIH (R21AG080410, R35AG072362, U01NS128913, P30AG062422, R01AG059183, and UG3AG090679) and Global Brain Health Institute. GS‐B receives funding from the Ministerio de Ciencia e Innovacion, Spanish Research Agency, PID2020‐119556RA‐I00. MM‐A receives funding from Alzheimer's Association Research Fellowship Program (AARF‐23‐1141384). OGR receives funding from the Alzheimer's Association Research Fellowship Program (2019‐AARF‐644568), from Instituto de Salud Carlos III (PI19/00117) and from the Spanish Ministry of Science Innovation and Universities (Juan de la Cierva programme IJC2020‐043417‐I). JDG DG is supported by the Spanish Ministry of Science and Innovation (RYC‑2013‑13054). JDG has also received research support from the EU/EFPIA Innovative Medicines Initiative Joint Undertaking AMYPAD (grant agreement 115952), EIT Digital (Grant 2021), and from Ministerio de Ciencia y Universidades (grant agreement RTI2018‑102261). KP receives funding from the NIH (U01NS128913, UH3NS105557, and R35AG072362), Quest Diagnostics, the Global Brain Health Institute, the Merck Foundation, and the Rainwater Charitable Foundation.

## CONFLICT OF INTEREST STATEMENT

Anna Brugulat‐Serrat, Elena Tsoy, Marta Milà‐Alomà, Juan Domingo Gispert, Joel H Kramer, and Katherine L Possin reported they have nothing to disclose. O.G.R. receives research funding from F. Hoffmann‐La Roche Ltd and has given lectures in symposia sponsored by Roche Diagnostics, S.L.U. G.S.‐B. worked as a consultant for Roche Farma, S.A. J.D.G. receives research funding from Roche Diagnostics and GE Healthcare, has given lectures at symposia sponsored by Biogen and Philips, and is currently an employee of AstraZeneca. Author disclosures are available in the Supporting Information.

## CONSENT STATEMENT

The EPAD LCS protocol and materials are submitted to the Independent Ethics Committee or other relevant ethical review board for written approval as required by local laws and regulations. A copy of approval is required by the University of Edinburgh as Sponsor before the study commences at each site. The study is designed and conducted in accordance with the guidelines for Good Clinical Practice (GCP), and with the ethical principles as proclaimed in the Declaration of Helsinki. All participants are required to provide written informed consent prior to participation in any research activities laid out in the EPAD LCS protocol.

## Supporting information



Supporting information

Supporting information
