# Supplementary material for: Detecting early memory changes in preclinical Alzheimer's disease using TabCAT favorites test: Data from the European Prevention of Alzheimer's Disease (EPAD) cohort
Source: Alzheimers Dement. 2026 Feb 13;22(2):e71035. doi: 10.1002/alz.71035 (PMC12902892; doi:10.1002/alz.71035)
Supplement: Supplementary file 1 — Supporting information [file ALZ-22-e71035-s002.docx]

**Supporting Information**

**Detecting early memory changes in preclinical Alzheimer's disease using TabCAT Favorites Test: data from the European Prevention of Alzheimer's Disease (EPAD) cohort**

Anna Brugulat-Serrat, Elena Tsoy, Gonzalo Sánchez-Benavides, Marta Milà-Alomà, Leslie Gaynor, Oriol Grau-Rivera, Juan Domingo Gispert, Joel H Kramer, Katherine L Possin, for the European Prevention of Alzheimer's Disease (EPAD) Consortium

| **Table S1. Associations between Favorites-TC and RBANS-MI and AT stages** | | | | | | | | | |  |  |  |  |  |
| --- | --- | --- | --- | --- | --- | --- | --- | --- | --- | --- | --- | --- | --- | --- |
|  | **A+T-** | | | |  | **A+T+** | | | |  | **A-T+** | | | |
| **Ref. group= A-T-** | **β** | **OR** | **95% CI of OR** | ***p*** |  | **β** | **OR** | **95% CI of OR** | ***p*** |  | **β** | **OR** | **95% CI of OR** | ***p*** |
| **Multinomial regression with Favorites-TC** | | | | | | | | | | | | | | |
| **Age** | 0.04 | 1.04 | 1.01 – 1.07 | 0.019* |  | 0.16 | 1.18 | 1.09 – 1.26 | <.001* |  | 0.10 | 1.00 | 0.94 – 1.07 | 0.935 |
| **Sex^a^** | 0.08 | 1.09 | 0.75 – 1.58 | 0.659 |  | 0.15 | 1.16 | 0.53 – 2.55 | 0.715 |  | -0.38 | 0.68 | 0.35 – 1.30 | 0.245 |
| **Educational level^a^** | | | | | | | | | | | | | | |
| Elementary | -0.34 | 0.71 | 0.38 – 1.35 | 0.296 |  | 1.63 | 5.13 | 0.59 – 44.19 | 0.137 |  | 17.97 | 6.43 | 3.02 – 13.69 | <.001* |
| Secondary | -0.61 | 0.54 | 0.29 – 1.03 | 0.060 |  | 0.69 | 2.01 | 0.20 – 19.66 | 0.552 |  | 17.96 | 6.32 | 2.99 – 13.35 | <.001* |
| Graduate | -0.42 | 0.66 | 0.37 – 1.17 | 0.152 |  | 1.41 | 4.09 | 0.48 – 34.68 | 0.196 |  | 18.14 | 7.56 | 7.55 – 7.5.6 | 0.365 |
| ***APOE*-ε4 status^a^** | -0.82 | 0.44 | 0.31 – 0.64 | <.001* |  | -2.34 | 0.09 | 0.04 – 0.24 | <.001* |  | -0.53 | 0.59 | 0.31 – 1.12 | 0.105 |
| **Favorites-TC** | 0.00 | 1.00 | 0.96 – 1.04 | 0.976 |  | -0.80 | 0.92 | 0.85 – 0.99 | 0.047* |  | 0.00 | 1.00 | 0.94 – 1.06 | 0.935 |
|  | **A+T-** | | | |  | **A+T+** | | | |  | **A-T+** | | | |
| **Ref. group= A-T-** | **β** | **OR** | **95% CI of OR** | ***P*** |  | **β** | **OR** | **95% CI of OR** | ***p*** |  | **β** | **OR** | **95% CI of OR** | ***p*** |
| **Multinomial regression with RBANS-MI** | | | | | | | | | | | | | | |
| **Age** | 0.04 | 1.04 | 1.00 – 1.07 | 0.013* |  | 0.19 | 1.21 | 1.12 – 1.29 | <.001* |  | 0.09 | 1.10 | 1.04 – 1.16 | <.001* |
| **Sex^a^** | -0.09 | 0.91 | 0.62 – 1.31 | 0.606 |  | -0.30 | 0.74 | 0.34 – 1.61 | 0.449 |  | 0.39 | 1.49 | 0.78 – 2.83 | 0.226 |
| **Educational level^a^** | | | | | | | | | | | | | | |
| Elementary | -0.32 | 0.73 | 0.38 – 1.38 | 0.331 |  | 1.66 | 5.26 | 0.62 – 44.44 | 0.127 |  | 17.93 | 6.13 | 2.88 – 13.03 | <.001* |
| Secondary | -0.61 | 0.54 | 0.29 – 1.03 | 0.063 |  | 0.72 | 2.06 | 0.21 – 19.87 | 0.532 |  | 17.95 | 6.27 | 2.89 – 13.21 | <.001* |
| Graduate | -0.42 | 0.66 | 0.37 – 1.17 | 0.152 |  | 1.29 | 3.65 | 0.44 – 30.08 | 0.230 |  | 18.13 | 7.51 | 7.51 – 7.52 | <.001* |
| ***APOE*-ε4 status^a^** | -0.82 | 0.44 | 0.30 – 1.02 | <.001* |  | -2.34 | 0.09 | 0.04 – 0.24 | <.001* |  | -0.51 | 0.59 | 0.32 – 1.13 | 0.115 |
| **RBANS-MI** | 0.01 | 1.00 | 0.99 – 1.02 | 0.499 |  | 0.01 | 1.01 | 0.97 – 1.04 | 0.734 |  | -0.01 | 0.99 | 0.96 – 1.01 | 0.349 |
| Notes: Independent multinomial logistic regressions examining the associations between episodic memory performance and AT stages, covaried by age, sex, education level, and *APOE*-ε4 status.  ^a^Reference groups: *APOE*-ε4 status= ε4 non-carriers; Educational level= postgraduate; Sex= female.  ^*^P < .05 | | | | | | | | | | | | | | |

**Table S2. Associations between Favorites-TC and RBANS-MI and AT stages**

|  | | | | | | | | | |
| --- | --- | --- | --- | --- | --- | --- | --- | --- | --- |
|  | **A+T-** | | | |  | **A+T+** | | | |
| **Ref. group= A-T-** | **β** | **OR** | **95% CI of OR** | ***p*** |  | **β** | **OR** | **95% CI of OR** | ***p*** |
| **Multinomial regression with Favorites-TC** | | | | | | | | | |
| **Age** | 0.05 | 0.00 | 1.02 – 1.08 | 0.003* |  | 0.16 | 1.17 | 1.09 – 1.26 | <.001* |
| **Sex^a^** | 0.94 | 0.57 | 1.78 – 3.70 | <.001* |  | -0.24 | 0.79 | 0.37 – 1.69 | 0.538 |
| **Educational level^a^** | | | | | | | | | |
| Elementary | -0.48 | 0.62 | 0.33 – 1.16 | 0.131 |  | 0.79 | 2.22 | 0.43 – 11.33 | 0.338 |
| Secondary | -0.62 | 0.54 | 0.29 – 0.99 | 0.049* |  | -0.01 | 0.98 | 0.17 – 5.79 | 0.988 |
| Graduate | -0.49 | 0.09 | 0.35 – 1.08 | 0.089 |  | 0.70 | 2.02 | 0.40 – 10.12 | 0.392 |
| ***APOE*-ε4 status^a^** | 0.94 | 2.57 | 1.78 – 3.69 | <.001* |  | 2.34 | 10.38 | 4.23 – 25.41 | <.001* |
| **Favorites-TC** | 0.00 | 1.00 | 0.97 – 1.04 | 0.813 |  | -0.08 | 0.92 | 0.86 – 0.99 | 0.030* |
|  | **A+T-** | | | |  | **A+T+** | | | |
| **Ref. group= A-T-** | **β** | **OR** | **95% CI of OR** | ***p*** |  | **β** | **OR** | **95% CI of OR** | ***p*** |
| **Multinomial regression with RBANS-MI** | | | | | | | | | |
| **Age** | 0.04 | 1.04 | 1.01 – 1.07 | 0.007* |  | 0.18 | 1.20 | 1.12 – 1.29 | <.001* |
| **Sex^a^** | -0.16 | 0.85 | 0.59 – 1.23 | <.001* |  | -0.29 | 0.75 | 0.35 – 1.63 | 0.471 |
| **Educational level^a^** | | | | | | | | | |
| Elementary | -0.37 | 0.69 | 0.37 – 1.30 | 0.249 |  | 1.28 | 4.40 | 0.52 – 36.96 | 0.172 |
| Secondary | -0.69 | 0.50 | 0.27 – 0.94 | 0.032 |  | 0.70 | 2.01 | 0.21 – 19.41 | 0.546 |
| Graduate | -0.50 | 0.61 | 0.34 – 1.07 | 0.081 |  | 1.26 | 3.54 | 0.43 – 29.12 | 0.240 |
| ***APOE*-ε4 status^a^** | 0.87 | 2.39 | 1.65 – 3.45 | <.001* |  | 2.32 | 10.17 | 4.14 – 24.97 | <.001* |
| **RBANS-MI** | 0.01 | 1.01 | 0.994 – 1.02 | 0.242 |  | 0.01 | 1.01 | 0.97 – 1.04 | 0.777 |
| Notes: Independent multinomial logistic regressions examining the associations between episodic memory performance and AT stages, covaried by age, sex, education level, and *APOE*-ε4 status.  ^a^Reference groups: *APOE*-ε4 status= ε4 non-carriers; Educational level= postgraduate; Sex= female.  ^*^P < .05 | | | | | | | | | |

| **Table S3. Associations between Favorites-TC and AT stages additionally adjusted by HVa** | | | | | | | | | |
| --- | --- | --- | --- | --- | --- | --- | --- | --- | --- |
|  | **A+T-** | | | |  | **A+T+** | | | |
| **Ref. group= A-T-** | **β** | **OR** | **95% CI of OR** | ***p*** |  | **β** | **OR** | **95% CI of OR** | ***p*** |
| **Multinomial regression with Favorites-TC** | | | | | | | | | |
| **Age** | 0.04 | 1.04 | 1.01 – 1.071 | 0.015* |  | 0.178 | 1.19 | 0.85 – 0.99 | <.001* |
| **Sex^a^** | -0.13 | 0.88 | 0.60 – 1.27 | 0.486 |  | 0.132 | 1.14 | 0.49 – 2.63 | 0.757 |
| **Educational level^a^** | | | | | | | | | |
| Elementary | -0.41 | 0.66 | 0.35 – 1.25 | 0.664 |  | 1.58 | 4.86 | 0.56 – 42.44 | 0.152 |
| Secondary | -0.67 | 0.51 | 0.27 – 0.97 | 0.515 |  | 0.70 | 2.01 | 0.20 – 20.23 | 0.552 |
| Graduate | -0.49 | 0.61 | 0.34 – 1.08 | 0.611 |  | 0.15 | 4.29 | 0.50 – 36.82 | 0.184 |
| ***APOE*-ε4 status^a^** | 0.87 | 0.34 | 1.64 – 3.44 | <.001* |  | 2.41 | 11.19 | 4.44 – 28.18 | <.001* |
| **HVa** | 0.00 | 1.00 | 1.00 – 1.01 | 0.837 |  | 0.00 | 1.00 | 1.00 – 1.001 | 0.029* |
| **Favorites-TC** | 0.00 | 1.00 | 0.96 – 1.04 | 0.996 |  | -0.09 | 0.92 | 0.85 – 0.99 | 0.033* |
| Notes: Independent multinomial logistic regressions examining the associations between episodic memory performance and AT stages, covaried by age, sex, education level, *APOE*-ε4 status, and HVa.  ^a^Reference groups: *APOE*-ε4 status= ε4 non-carriers; Educational level= postgraduate; Sex= female.  ^*^P < .05 | | | | | | | | | |

**Table S4. ROC curve coordinates by Favorites-TC cutoffs**

| **Favorites-TC cutoff** | **Sensivity** | **Specificity** | **Youden Test** |
| --- | --- | --- | --- |
| 0 | 0.000 | 0.983 | 0.000 |
| 2 | 0.000 | 0.964 | -0.017 |
| 3 | 0.000 | 0.951 | -0.036 |
| 4 | 0.086 | 0.945 | 0.037 |
| 5 | 0.114 | 0.932 | 0.060 |
| 6 | 0.114 | 0.919 | 0.047 |
| 7 | 0.114 | 0.902 | 0.033 |
| 8 | 0.200 | 0.874 | 0.102 |
| 9 | 0.286 | 0.825 | 0.160 |
| 10 | 0.343 | 0.797 | 0.168 |
| 11 | 0.429 | 0.756 | 0.226 |
| 12 | 0.514 | 0.724 | 0.270 |
| 13 | 0.714 | 0.692 | 0.406 |
| 15 | 0.743 | 0.639 | 0.382 |
| 16 | 0.800 | 0.573 | 0.373 |
| 17 | 0.857 | 0.504 | 0.361 |
| 18 | 0.857 | 0.432 | 0.289 |
| 19 | 0.886 | 0.378 | 0.264 |
| 20 | 0.886 | 0.306 | 0.192 |
| 21 | 0.914 | 0.229 | 0.144 |
| 22 | 0.943 | 0.126 | 0.069 |
| 23 | 0.943 | 0.060 | 0.003 |
| 24 | 1.000 | 0.024 | 0.024 |

**Figure S1. Progression to CDR>0 by Favorites-TC optimal cutoff**
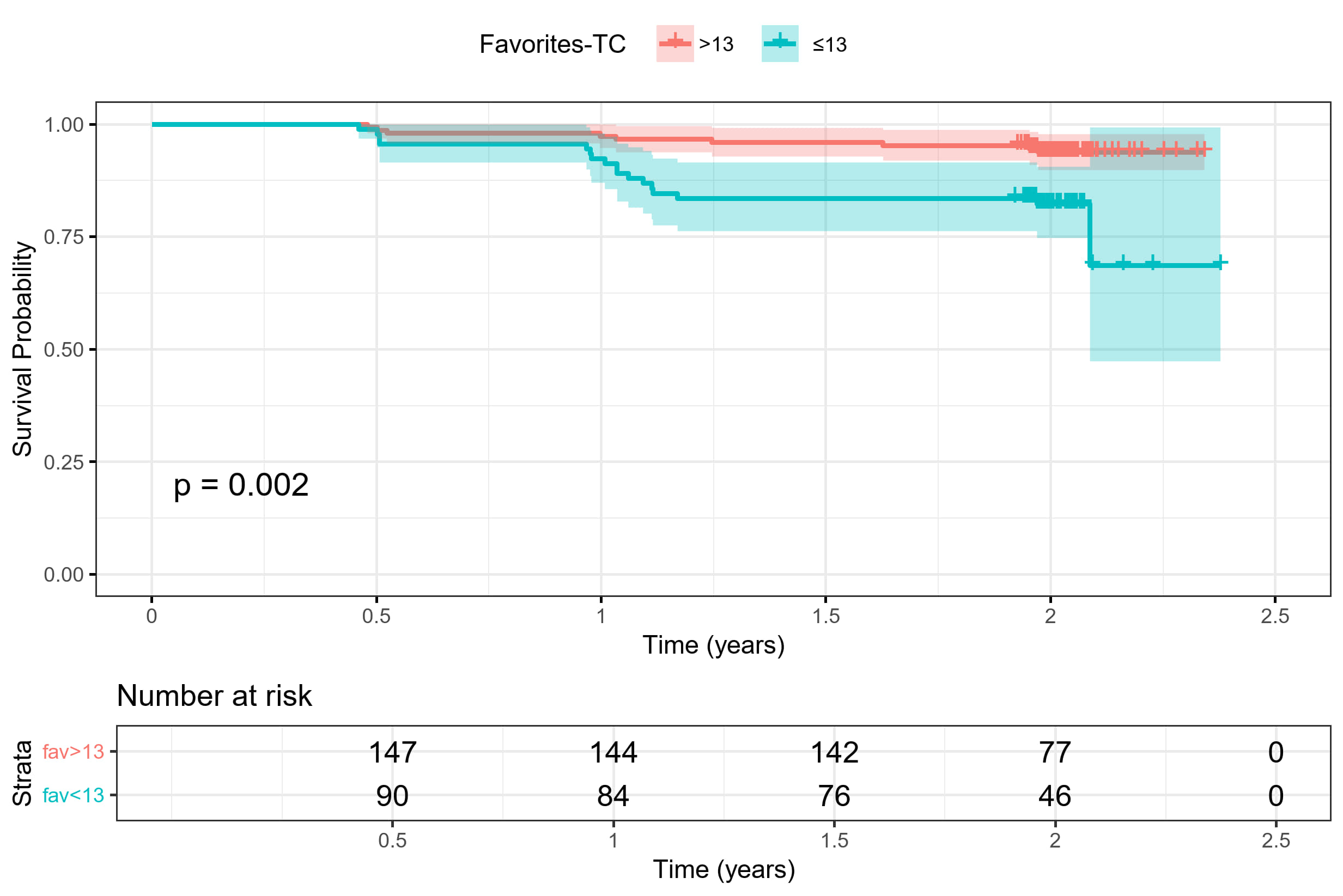


Note: Survival curves are shown in red Favorites-TC>13 and in blue for Favorites-TC≤13 and are adjusted for age at first visit, sex, and education level.
